# Making sense of conflicting messages of multiracial identity: a systematic review

**DOI:** 10.3389/fpsyg.2024.1307624

**Published:** 2024-04-25

**Authors:** Tatiana I. Zamora, Amado M. Padilla

**Affiliations:** Graduate School of Education, Stanford University, Stanford, CA, United States

**Keywords:** multiracial, adolescents, socialization, family, peers

## Abstract

**Background:**

Ethnic-racial identity (ERI) development refers to how individuals’ experiences, beliefs, and attitudes influence understanding of ethnic-racial group membership. Messages about race, from multiple ecosystems, influence identity development and how individuals come to form their ERI. There has been a shift in ERI research to focus on Multiracial populations, however, most of the research focus is on Black/white biracial and general, non-specified Multiracial populations. The ERI development process and experience for persons of other Multiracial backgrounds (e.g., AfroLatinx or AsianBlack) is not as extensively studied. This systematic literature review aims to elucidate the existing conceptualization of Multiracial ERI development for non-Black/white biracial and general Multiracial populations in the United States.

**Methods:**

A comprehensive search strategy was employed across multiple academic databases to identify relevant studies based on explicit inclusion criteria. The initial search resulted in 1,846 articles, but when only Black/white biracial and non-specified general Multiracial studies were eliminated from this review, only 18 articles met the criteria for inclusion.

**Results:**

Common themes emerged from the reviewed literature, including the importance of spaces, conflicting social messages directed at Multiracial individuals, and coping responses used by Multiracial individuals when faced with challenges by family members and peers regarding their multiracial identity.

**Discussion:**

The findings underscore the need for a more nuanced exploration of ERI development among diverse Multiracial populations. Understanding the unique strengths, experiences, and challenges of different Multiracial populations beyond the Black-white biracial paradigm is essential for understanding ERI development across and between different Multiracial populations in today’s world.

## 1 Introduction

The population of the United States is increasingly becoming more diverse, including a significant increase in the number of individuals who identify as Multiracial (Atkin et al., 2022). In line with this demographic shift, research should reflect the changing demographic profile of the country. Because of the ubiquitous nature of race and ethnicity in the United States (Wilkerson, 2020), it is critical to understand the varied processes through which Multiracial youth learn about race and ethnicity and how this shapes their understandings of themselves, and their relationships with others in the world around them (Burke and Kao, 2011). The purpose of this systematic literature review is to better understand the increasing complexity of ethnic racial identity (ERI) development of Multiracial individuals.

In this paper, the term *Multiracial* is used to denote individuals whose parents come from two or more ethnic or racial backgrounds. The definition encapsulates *biracial* individuals, which implies individuals whose heritage originates from two distinct ethnic or racial backgrounds, and the myriad backgrounds that reflect the growing racial diversity in this country.

### 1.1 Multiracial identity in the United States

Although race and ethnicity are inherently social constructs (Omi and Winant, 2014), they play an important role in one’s identity development. *Race* is a social construct based on phenotypic characteristics, such as skin color or hair texture, and a shared history based on these superficial features (Gonzales-Backen, 2013). *Ethnicity* refers to one’s ancestry, culture, language, and geographic origin (Perez and Hirschman, 2009). In social settings and in research scholarship, both race and ethnicity are often used interchangeably, thus the use of the term *ethnic-racial identity (ERI)* when considering how these social constructs play a role in a person’s identity development. ERI is considered a “multidimensional, psychological construct that reflects the beliefs and attitudes individuals have about their ethnic-racial group memberships, as well as the processes by which these beliefs and attitudes develop over time” (Umaña-Taylor et al., 2014, p. 23). This definition encapsulates both ethnic background and racialized experiences in understanding an individual’s group membership.

Until recently, Multiracial individuals were expected to identify with only one of their racial/ethnic backgrounds and most generally this conformed to the group that the Multiracial person resembled most in phenotype when filling out demographic forms and other paperwork. This changed with the 2000 U.S. Population Census where Multiracial individuals were allowed to self-select more than one race category to indicate their ERI (Jones and Smith, 2001). Historically, Multiracial individuals have been categorized and perceived as monoracial, with the focus on the more stigmatized and lower status racial group, such as Black, Latinx, or Asian (Daniel, 1996). This categorization bias of *hypodescent* was motivated by the white majority upholding their dominant status and power in the U.S. society (Davis, 1991; Ho et al., 2013; Krosch et al., 2013).

Beginning with the 2000 Census, the number of people reporting being Multiracial has increased substantially (Jones et al., 2021). The 2000 Census was the first time participants were allowed to “mark one or more races” when completing the Census, resulting in 2.4% of the US population indicating that they identified as Multiracial. In 2010, 2.9% of the US population identified as Multiracial. From the 2010 to 2020 Census, the percentage of respondents who reported more than one race increased dramatically and more than other single racial groups, with 10.2% of the population identifying as Multiracial.

Despite the increase in the number of people identifying as Multiracial, this does not preclude teachers and peers from using the notion of hypodescent to classify a Multiracial person according to their salient physical characteristics such as skin color. However, Multiracial individuals also face a unique predicament in which they are subject to distinct stereotypes about being Multiracial (Skinner et al., 2020). This includes stereotypes about being unusual or unique and not fitting in or belonging to their racial groups (Miville et al., 2005; Skinner et al., 2020). Further, Multiracial individuals are often subjected to microaggressions including being excluded, being objectified, assumed to be monoracial or from a different racial group, having their experience as a Multiracial person denied, and their identity pathologized as anomalous (Johnston and Nadal, 2010). Importantly, the marginalization often comes from members of both parental racial groups (e.g., white-Asian) that the biracial person identifies with.

### 1.2 Ethnic-racial identity

For Multiracial individuals, ERI reflects the developmental process of experiencing and understanding both ethnic-racial groups, and the actual beliefs and attitudes about dual ethnic-racial group membership (Umaña-Taylor et al., 2014). Umaña-Taylor et al. (2014) explore five main aspects in which one considers their ERI: (1) exploration, (2) resolution, (3) centrality, (4) positive affect, and (5) public regard. *Exploration* is the way in which one thinks about and explores or considers what group membership personally means to them. *Resolution* is the commitment to one’s ERI in one’s daily life. *Centrality* is the importance of one’s group membership to their self-concept and overall wellbeing. *Positive affect* refers to the feeling of belonging to an ethnic-racial group. Lastly, *public regard* includes the beliefs an individual has about how others view their ethnic-racial group. These aspects all influence the way an individual may self-categorize themself, as well as how they may or may not identify with their ethnic/racial heritage. Furthermore, content and process are two other components of ERI development. *Content* includes attitudes and beliefs about one’s own and other ethnic/racial groups, while *process* includes the different ways in which individuals explore and identify their ERI. Because of this, ERI can be considered the process in identifying one’s ERI, as well as the self-categorization that one chooses. This also captures that ERI can change over time.

Although race is not a fixed concept, it stabilizes as a social construct for monoracial individuals, once committed to their identity. On the other hand, research shows that ERI may be fluid, both developmentally across the lifespan, such as Multiracial individuals who switch their self-identification throughout adolescence by adding a racial category or consolidating their identity by subtracting an ERI (Hitlin et al., 2006). Situational fluidity includes cognitive frame switching (McFarland and Fingerhut, 2011) and developing flexible strategies to fit in multiple social groups (Miville et al., 2005). Fluidity in an adolescent’s racial identity is influenced by parents and other family members, or by extra-familial situations such as not having contact with one of their birth parents, thus not being able to fully explore that part of their identity, or through a realization that they have been critical of their own ethnic-racial group, such as going through a period of militancy against white individuals, before realizing that they also belong to that group (Miville et al., 2005). ERI self-categorization is also influenced by peers: either by peers’ categorization of them (Booth et al., 2022), or experiencing racial diversity among friends and classmates (Echols et al., 2017). Essentially, the fluidity of ethnic-racial identity is unique to Multiracial individuals as they strive to belong in a racial space (s) they do and do not fully occupy due to monoracism.

Other theories on Multiracial ethnic-racial identity have focused on one of four views: (1) the problem approach, (b) the equivalent approach, (3) the variant approach, and (4) the ecological approach. The *problem approach* considers Multiracial identity to be a separate entity that is problematic for the individual (Rockquemore et al., 2009). The problematic approach represents a deficit perspective that solely focuses on the dilemmas and challenges a person faces due to their identity. The *equivalent approach* stems from the hypodescent categorization bias, in which multiracial individuals are perceived to be members of the lower status racial group. The var*iant approach* considers how a Multiracial identity differs from a monoracial identity development. Lastly, the *ecological approach* considers how one’s identified social position depends upon the context or environment they are currently occupying. These four different theoretical perspectives of Multiracial identity require that we consider how these different theories contribute to our understanding of the Multiracial experience.

An identity matrix, a concept borrowed from Collins J. F.’ (2000) insights on Black feminist thought (Collins P. H., 2000), demonstrates the complexity of a multiracial experience as one’s various selves interact with one another (Brunsma et al., 2013). This matrix allows one’s identity to correspond and shift depending on the context the Multiracial person is in at any one time. Furthermore, the matrix recognizes the complexities and fluidity of a Multiracial identity, unlike other theories, by allowing Multiracial individuals to express how they view themselves (e.g., Black, white, biracial) across a variety of variables, including racial identity, skin color, hair texture, gender, and geographic region. This exemplifies how a Multiracial identity may not draw from a singular Multiracial experience and is influenced through cross-racial interactions, friendships, intimacies, and solidarities. According to this approach, Multiracial individuals are afforded unique agency in which they may benefit from privilege and suffer from discrimination depending on the physical, political, social, or cultural environment they are in at any one time.

Kerwin and Ponterotto’s (1995) biracial identity development model proposes age-based developmental markers to illustrate how children and adolescents become aware of racial differences. This model is centered on the view that as biracial and Multiracial children come of school age and progress to adolescence, they are likely exposed to racism as well as experience social pressure from peers to identify with a specific ethnic or racial group. Essentially, the Multiracial adolescent is pressured to pick a side and to identify with that group. Consistent with this model, Multiracial students report experiencing prejudice and microaggressions in relation to their identity (ies) in various school and community settings (Brackett et al., 2006).

### 1.3 Ethnic-racial socialization

Ethnic-racial socialization (ERS) is the developmental process in which children learn about the behaviors, values, perceptions, and attitudes of an ethnic-racial group (Phinney and Rotheram, 1987). Most of the literature assumes that ERS is a parenting practice where parents transmit verbal and nonverbal messages on race and ethnicity, as well as sharing about interracial and interethnic interactions with the outcome of learning *coping skills* to combat the stress due to racism (Hughes et al., 2006; Berkel et al., 2010). These coping skills include parents communicating with youth about racial dynamics, learning how to recognize and address discrimination, and developing a positive and healthy ERI (Hughes et al., 2006).

More recent empirical and theoretical scholarship has focused on how schools (Aldana and Byrd, 2015; Byrd, 2015; Saleem and Byrd, 2021), neighborhoods (Moje and Martinez, 2007; Witherspoon et al., 2021), and peers (Rivas-Drake et al., 2017; Santos et al., 2017; Burkholder et al., 2021) can also be viewed as socializing agents. Across all these different contexts and interactions, youth are socialized about race and ethnicity through verbal and nonverbal messages, including stories from parents, teachers and peers about everyday interactions and youth’s observations of others’ behaviors or conversations (Hughes et al., 2006; Watford et al., 2021).

Importantly, ERS research considers how monoracial parents do not have the same ethnic-racial group classification as their Multiracial children since their children have a combination of the parents’ ethnic-racial groups; this presents unique challenges for parents when discussing race with children (Rondilla et al., 2017). Further, this can be especially problematic since most ERS research assumes family members share a single racial identity and heritage (Samuels, 2009). Parents of Multiracial children have multiple identities that they can foster in their children, including the biological mother’s ERI, biological father’s ERI, and a child with a Multiracial identity (Atkin and Yoo, 2019). Because of these differing ethnic-racial backgrounds and experiences, parents may have different attitudes, approaches, and practices to socializing their Multiracial child(ren) (Gonzales-Backen, 2013; Csizmadia et al., 2014). Additionally, parents and caregivers are in a unique position in that they are socializing Multiracial children without themselves having experienced their children’s Multiracial reality (Rockquemore and Laszloffy, 2005; Rollins and Hunter, 2013). Furthermore, when one parent is white, they do not understand the experience of being a person of color and may experience difficulty helping their children make sense of racialized experiences (Rauktis et al., 2016). Parents from different cultures and ethnic-racial backgrounds may choose to integrate multiple ethnic-racial backgrounds into their family dynamics and must learn about these different backgrounds and navigate differences between these ethnic-racial positions.

The exploration and commitment to one’s ethnic-racial identity may partly be influenced by ethnic-racial socialization practices in both family and school contexts. The opportunity to learn about one’s ethnic-racial heritage has been shown to be positively associated with youths’ exploration and commitment to their identity (Byrd and Legette, 2022). Whether a student is either still exploring their Multiracial identity or has committed to an identity, this has implications for how the young person interprets their racial reality and the world around them. Accordingly, it is important to consider how this identity development process is not universal for all Multiracial students, despite sharing a similar Multiracial experience.

### 1.4 Black-white biracial experience

Scholarship on Multiracial identity theories has largely been based on the experiences of Black-white biracial individuals. Biracial Black-white individuals have reported more prejudice across different contexts, both affirming and threatening, compared to Black individuals (Rozek and Gaither, 2021). Biracial Black-white individuals experience stereotypes and threats consistent with Black stereotypes, such as being good at sports and bad at academic subjects like math, but they also experience additional stereotypes because of being biracial, such as not belonging to either group and not actually being Black or “Black Enough” in their behavior (Franco et al., 2016; Rozek and Gaither, 2021).

Furthermore, biracial black-white individuals also experience racial invalidation, as early as elementary school (Franco et al., 2016). Racial invalidation can be accusations of racial inauthenticity, imposition of racial categories, and forcing individuals to choose one aspect of their identity (Rockquemore and Brunsma, 2002; Buckley and Carter, 2004; Gillem and Thompson, 2004). This invalidation happens mostly at school, or while spending time with friends and Black community members and filling out official survey forms requiring demographic information such as mono-racial identification.

Although scholarship that focuses exclusively on mixed White-Black persons is valuable and insightful, it does not fully capture the unique and lived experiences of different Multiracial populations. In her pioneering work with Multiracial Asian Americans and other mixed racial persons, Root (1992, 1997) pointed to the diversity inherent in Multiracial individuals and argued that it was important to disaggregate the multiple groupings in research to better grasp how racism operates both at the societal (macrolevel) and microlevel within each racial group to understand identity development and intergroup relations. Following Root’s lead, this paper focuses exclusively on the research literature of other racially mixed people (e.g., Latinx-White; Asian-Latinx) to examine similarities and differences in how other racially mixed categories of people experience their Multiraciality and how this is shaped by peers and others in one’s social sphere of influence. The goal of this paper is to create a broader understanding of the Multiracial experiences of people (e.g., white and Latinx, Asian and Black) who reside in the United States.

## 2 Theoretical framework

### 2.1 Ecological systems

Ecological systems consider an individual’s relationship with their environment and how their experience is shaped by individual traits interacting with their environment. Bronfenbrenner’s (1994) ecological model of human development contains five systems that vary in proximity to the individual. This model is typically depicted by five concentric circles in which the individual is at the center of these circles. The surrounding concentric circles include the microsystem, mesosystem, exosystem, macrosystem, and chronosystem.

The microsystem is the most proximal environment to the individual (Bronfenbrenner, 1994). The microsystem includes people that the individual encounters on a daily basis such as family, classmates, friends, and both direct interactions and interpersonal relationships with teachers and peers in school. The mesosystem describes the interrelationships between different microsystems (Bronfenbrenner, 1994). It includes the interplay of those who exist in one’s microsystem. The exosystem considers the interactions between different contexts (Bronfenbrenner, 1994). One of these contexts must not include the individual, but still has some influence over the individual’s experience, such as mass media. The macrosystem describes the greater context in which the child/person lives. This includes the characteristics, belief systems, bodies of knowledge, and cultures of the micro-, meso-, and exosystems, such as socioeconomic status and cultural ideologies. Lastly, the chronosystem considers how change in time affects the characteristics of an individual and the characteristics in the environment in which the person lives. This generally includes major world events, such as increasing focus on multiculturalism and global migration.

By using an ecological lens, we can shift from individual-level processes to studying the settings that produce these ethnic-racial dynamics (Hughes et al., 2016). Identity development is a process that is produced in space and time, meaning it is influenced by other socializing agents and settings (Moje and Martinez, 2007). Therefore, by knowing what messages youth and adolescents receive and where these messages come from, we can begin to understand how different environments influence their identity development. We can think of Bronfenbrenner’s model as a framework that interrogates the various levels of influence on development, and how these various levels intertwine with one another. Understanding both the differences and overlaps between the various levels can help us better understand how different environments interact and influence development.

### 2.2 Strengths-based approach

The concept of a “cultural home” emphasizes the emotional meaning attached to the historical continuity and cognitive frame of reference for social interaction within a common history and shared activities and traditions along with collective support (Vivero and Jenkins, 1999). Research on the Multiracial experience, including identity development, suggests that the early-life immersion in more than one culture leads to feelings of being different from one’s parents and other family members and not feeling like the person fits into a fixed racial or ethnic category (Vivero and Jenkins, 1999; Navarrette and Jenkins, 2011). This approach, along with many identity theories and approaches, attempts to center and emphasize the challenges of being Multiracial. Rather than using a deficit-based lens to view the ethnic-racial identity development and experiences of Multiracial individuals, we utilize a strengths-based approach that shows that biracial individuals have a wealth of unique experiences that merit intense study for their social contributions to society and culture.

A strengths-based approach builds upon the individual’s strengths and promotive factors and sees the individual as resourceful, resilient, and unique (McCashen, 2017). Assets and resources are two types of promotive factors that can provide youth with individual and contextual attributes that are necessary for a positive and healthy development (Fergus and Zimmerman, 2005). Assets refer to positive factors, such as self-efficacy and self-esteem, that reside within individuals. Resources, on the other hand, refer to external or communal factors such as parental support, adult mentors, and youth programs that serve to enhance an individual’s sense of self as they generate strategies and solutions for the multiracial person to overcome adversity (e.g., racism) in their social context.

## 3 Current study

While there have been reviews focusing on ethnic-racial identity (Ream, 2023) and Multiracial populations (Charmaraman et al., 2014) these papers are valuable for their bibliometric methodology, however, few reviews exist that focus exclusively on the Multiracial experiences of Multiracial individuals. An exception is the work of Atkin and Yoo (2019) who conducted the first review on the familial racial socialization of Multiracial youth. In this paper Atkin and Yoo (2019) identified 21 peer reviewed quantitative (*N* = 7) and qualitative (*N* = 14) empirical papers. The reviewers noted that the majority of research concentrated on Black/white Multiracial children and the ways in which parents sought to socialize their children to one or the other or both of their racial backgrounds. A notable determinant of parents’ socialization strategy focused on the phenotype of the mixed race child(ren). Atkin and Yoo (2019) also pointed to the need to focus research on other ethno-racial mixed children since demographically these children represent a sizable portion of the U. S. population. Furthermore, other literature reviews have examined Black-white biracial identity development (Csizmadia et al., 2012; Dupree et al., 2015) and familial socialization practices (Green et al., 2021).

Thus, the goal of the current literature review is to deepen the psychological and social understanding of non-Black/biracial and Multiracial persons and to center these other identities, perspectives, and experiences in the broader field of socialization theory. Furthermore, while reviews have examined the Black-white biracial experience, there has not been a rigorous examination that focuses on other Multiracial groups (e.g., Latino-white, Asian-Latino). The typical research approach has been to lump these other Multiracial individuals with rich Multiracial backgrounds into a single category as if they shared common experiences despite their many differences. This selective procedure neglects and invalidates the experiences and backgrounds of other Multiracial groups and individuals (Atkin and Yoo, 2019). This review seeks to broaden our understanding of the ethnic-racial identity development and socialization of Multiracial individuals through a focus on empirical literature that centers specifically on the experiences of non-Black/white ethnically mixed background individuals, since Multiracial people with different ancestral backgrounds may be influenced and affected by different cultures, languages, and experiences. This will allow for a more nuanced understanding of Multiracial experiences. In this review our intent is to explore how Multiracial individuals navigate racially explicit messages when trying to come to grips with their own identity, while understanding the different messages that multiracial individuals receive across social contexts.

### 3.1 Methodology

Since Multiracial identity was first formally recognized and included in the 2000 U. S. Census, the literature in this review reflects Multiracial identity development from that year forward. This systematic review of the empirical research literature from 2000 to 2022 is directed at identifying texts that explicitly specify the racial/ethnic groups that were included in their study, this being either minoritized racial groups mixed with white, or dual-minoritized racial groups (e.g., Latinx-Asian, Asian-white, Native American, white). Research articles on white/Black mixed race are excluded from this systematic review because this category represents the most researched group to date. The overarching research question is:

*Research Question: How do the messages that non-Black/white biracial and Multiracial children and youth receive about race from family members, peers and others including Multiracial individuals and groups, influence their biracial identity development?*

This question aims to understand how non-Black/white biracial and Multiracial children and youth accommodate different positive and negative racial messages in order to adjust their ethnic-racial identity in order to achieve the benefits of being members of two racial groups.

### 3.2 Literature search and selection

Systematic reviews seek comprehensive knowledge of a given topic or question, while reducing bias in the selection and analysis of research studies on the topic of interest (Petticrew and Roberts, 2006). Research in this review was collected from the following five major electronic library databases: Google Scholar, PsycINFO, PsycARTICLES, Web of Science, and Sociological Abstracts. The review utilized the following search terms in various combinations:

*Multiracial, biracial, mixed race, Asian-white, Latinx-white, Native-white, minority-minority, Afro Latinx, Asian Latino, Black-Asian, ethnic-racial socialization, ethnic-racial identity development, school, peers, family, racial discrimination, racial messages, adolescent, and youth*

### 3.3 Inclusion and exclusion criteria

A text was included in this analysis if it: (1) was published between January 2000 and June 2022; (2) utilized empirical quantitative or qualitative methods and was published in a peer-reviewed journal; (3) specified collecting data on participants exclusively in the United States, and (4) explicitly stated the Multiracial population (s) that was studied. Article titles and abstracts were reviewed to determine whether a publication met these criteria.

**Figure 1:**
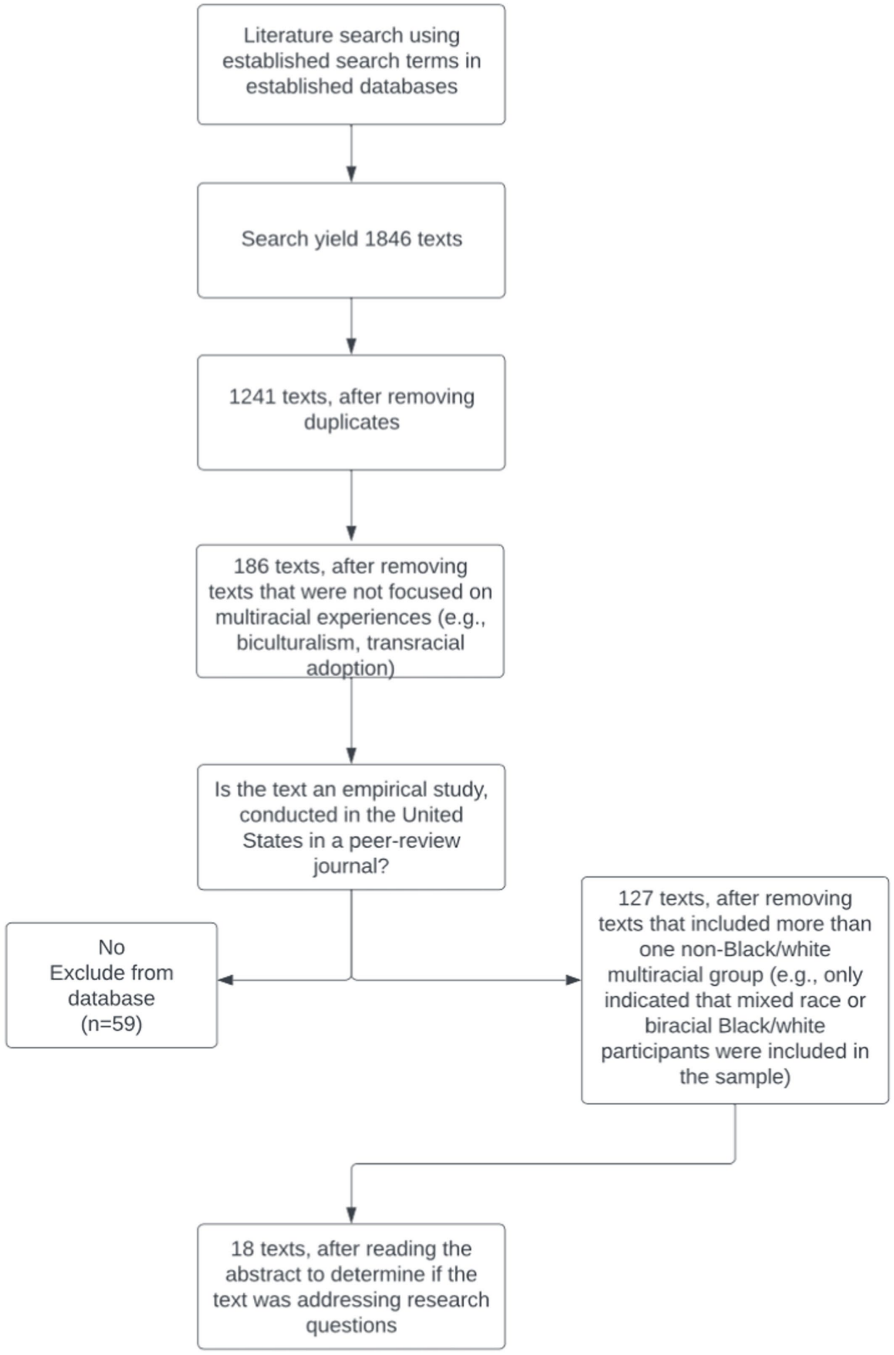
Flow chart with process of text selection.

Figure 1 illustrates the steps taken in selecting texts for this review. After gathering all the texts found using the above search terms and storing them in Zotero, duplicates were removed. The texts were then scanned to see whether they focused on Multiracial experiences. Studies that focused on transracial adoption or biculturalism were excluded. Theses, dissertations, book chapters, and other literature reviews were then excluded from the analysis. Lastly, if the title or abstract did not indicate the Multiracial population studied (e.g., Afro Latinx) or included more than one Multiracial group (e.g., only indicated that mixed race participants were included in the sample), then it was excluded because being classified as “Multiracial” erases the unique identities and experiences of that group (Table 1).

**Table 1:** Citations, titles, and relevant background information of research papers included in this systematic literature review.

Citation	Title	Sample and background	Methodology	Developmental period
García-Louis and Cortes (2023)^a^	Rejecting black and rejected black: AfroLatinx college students’ experiences with anti-AfroLatinidad	12 AfroLatinx College Students	Qualitative: Semi-Structured Interviews	Youth/Adolescent
Salas Pujols (2022)	‘It’s about the way i’m treated’: Afro-Latina black identity development in the third space	20 AfroLatina girls	Qualitative: Ethnographic Observations, In-depth Interviews	Youth/Adolescent
Allen et al. (2013)	Racial identity, phenotype, and self-esteem among biracial Polynesian/white individuals	84 Polynesian/White Adults	Quantitative: Survey	Adult
Castillo et al. (2020)	Black-Asian American identity: an exploratory study on how internalized oppression impacts identity development.	10 Black-Asian American adults	Qualitative: Grounded Theory Methods & Interviews	Adult
Collins J. F. (2000)	Biracial Japanese American identity: an evolving process	15 Biracial Japanese adults (ages 20-40)	Qualitative: Semi-Structured Interviews	Adult
Haywood (2017)	‘Latino spaces have always been the most violent’: Afro-Latino collegians’ perceptions of colorism and Latino intragroup marginalization	6 AfroLatinx college graduates (ages 22-41)	Qualitative: Semi-structured, Open-ended Interviews and 1 Focus Group	Adult
Hordge-Freeman and Loblack (2021)	“Cops only see the brown skin, they could care less where it originated”: Afro-Latinx perceptions of the #BlackLivesMatter movement	115 AfroLatinx Participants (10 who participated in interviews)	Qualitative: Open-ended Survey and Semi-Structured, In-Depth Interviews	Adult
Hordge-Freeman and Veras (2020)	Out of the shadows, into the dark: ethnoracial dissonance and identity formation among Afro-Latinxs	94 AfroLatinx Participants for Survey; 10 AfroLatinx Participants for Interviews	Mixed Methods: Quantitative and Qualitative Survey Responses; Semi-structured, In-depth Interviews	Adult
Iwasaki and Byrd (2010)	Cultural activities, identities, and mental health among urban American Indians with mixed racial/ethnic ancestries	25 multiracial Urban American Indians (ages 19-83)	Qualitative: Focus Groups	Adult
Jackson et al. (2013)	Mixed resilience: a study of multiethnic Mexican American stress and coping in Arizona	24 multiracial Mexican adults residing in Phoenix	Qualitative: Narrative Interviews	Adult
Jackson et al. (2019)	Parental ethnic-racial socialization in multiracial Mexican families	24 multiracial Mexican adults residing in Phoenix	Qualitative: Qualitative Secondary Analysis of Narrative Interviews	Adult
Khanna (2004)	The role of reflected appraisals in racial identity: the case of multiracial Asians	110 Asian-white Individuals	Quantitative: Survey	Adult
McCoy Gay et al. (2022)	“From all sides”: Black-Asian reddit communities identify and expand experiences of the multiracial microaggression taxonomy	Black-Asian Reddit Users (313 comments from Black-Asian Users across 15 subreddit threads)	Qualitative: Content Analysis	Adult
Romo (2011)	Between black and brown: Blaxican (Black-Mexican) multiracial identity in California	12 Black-Mexican “Blaxican” adults in California	Qualitative: In-Depth Interviews	Adult
Strmic-Pawl et al. (2022)	Asian-White mixed identity after COVID-19: racist racial projects and the effects on Asian multiraciality	46 Asian-white adults (ages 18–30)	Mixed Methods: Quantitative and Qualitative Survey, In-Depth Interviews	Adult
Kana’iaupuni and Liebler (2005)	Pondering poi dog: place and racial identification of multiracial native Hawaiians	2052 multiracial Native Hawaiian children (reported by their parents) from the 1990 US Census	Quantitative: Logistic Regression Analysis	Parent
Kohli et al. (2020)	'A drumbeat underneath the child'. Asian Indian mothers' perceptions of their multiethnic children's lived experiences	8 Asian Indian mothers with a multiracial child(ren)	Qualitative: Interviews	Parent
Liebler (2010)	Homelands and indigenous identities in a multiracial era	10,886 multiracial American Indian or Alaska Native from Census 2000 5% Public Use Microdata Sample (PUMS)	Quantitative: Logistic Regression Analysis	Parent

a Published online in 2020, published in its journal in 2023.

These criteria allowed for an in-depth analysis that emerges from what we already know from Black and white biracial and general Multiracial populations. Instead, these criteria allow a more focused review. The goal of this paper is to expand the understanding of specific and unique Multiracial experiences beyond those of Black/white mixed race participants.

### 3.4 Data analysis procedures

The initial search criteria yielded (*n* = 1,846) articles. Each article was screened for the criteria mentioned above and only articles that met the designated criteria were selected for inclusion in the next stage of the analysis. Once all the relevant articles (*n* = 18) were selected, they were sorted by developmental stages (i.e., youth/adolescent, adult, and parent). Adolescence is a period of transition for youth, and there are three main stages of adolescence: early adolescence (ages 11–14), middle adolescence (ages 15–17), and late adolescence/emerging adulthood (ages 18–25) (Arnett, 2000; Salmela-Aro, 2011). Therefore, the articles identified as youth and adolescents’ perspectives include high school and college students.

Adult perspectives include studies with participants who identified as 18+ across multiple stages of development (early adulthood, middle adulthood, and late adulthood). If the sample was not limited to college students, it was considered an adult perspective. Lastly, parent perspectives include parents reflecting on their Multiracial children. Typically, these studies examined monoracial parents raising multiracial children. Parents in these studies offered perspectives on how they are raising their child(ren), how they see their child(ren) developing, and how they report their child(ren)‘s ERI. These studies did not include any first-hand information on what the children think or how Multiracial parents discuss their parenting practices and beliefs. Once the texts were sorted, systematic coding was used to determine emerging themes across the articles.

The 18 articles that met the criteria for inclusion in this review varied by ethnic-racial background, methodology, and developmental perspectives. Most articles (*n* = 8) focused on Multiracial Latinx populations, with 6 of these specifically focusing on AfroLatinx participants. Six articles focused on Multiracial Asian populations, with two focused on Black-Asian backgrounds, two focused on Asian-white backgrounds, and two focused on the specific ethnic groups of biracial Japanese individuals and multiracial Asian-Indian. Lastly two articles focused on Multiracial American Indian and Alaska Native backgrounds, and 2 focused on Multiracial Native Hawaiian and Pacific Islander backgrounds.

The majority of articles employed a qualitative analysis (*n* = 14) in the study, whether the study was purely qualitative (*n* = 12) or mixed methods (*n* = 2). Qualitative approaches included interviews (e.g., open-ended, semi-structured, and life narrative), focus groups, content analysis and observations. Most of the qualitative studies employed interviews in their approaches, with supplementary data collection, such as a focus group or observations.

Quantitative studies (*n* = 6; for solely quantitative analysis, *n* = 3; quantitative mixed methods, *n* = 3) includes survey methodologies and logistic regression analyses. Two quantitative studies originating with Census data (Kana’iaupuni and Liebler, 2005; Liebler, 2010) come from a parental perspective. These two studies also are unique in that they focus on how home and place correlate to how parents are racially identifying their children.

## 4 Analysis

The analysis considers how different perspectives viewed messages and identity-development in different microsystems during development. The microsystems that were mentioned the most were immediate and extended family, educational spaces, and larger community spaces. In these spaces, socialization and identity-development were interrelated. Furthermore, developmental assets emerged within these unique microsystems across developmental periods.

### 4.1 Youth and adolescent perspectives

Articles (*n* = 2) that focus on youth and adolescent perspectives sampled AfroLatinx participants and emphasize how physical spaces, such as family gatherings, educational spaces, and student organizations, give different messages about their authenticity, belonging, and membership to their ethnic-racial groups.

Both articles emphasize the experiences with anti-AfroLatinidad, in which Blackness is rejected within the Latinx communities (Salas Pujols, 2022; García-Louis and Cortes, 2023). This includes experiencing microaggressions and a lack of discussion of Afro-Latinidad in family and school spaces. Rejection of Blackness from their Latinx community caused feelings of inferiority, undesirability, and not belonging to their community. The participants reported complex feelings stemming from their family rejection of their blackness, such as constant requests to relax their hair, and peers mocking their claimed Latinidad identity. This dual rejection led many individuals to fortify their experiences and assert their AfroLatinidad, rather than identify solely as Latino.

When students did not feel welcome in certain spaces, mainly Latinx spaces, they found refuge in Black/African American spaces or community spaces led by other AfroLatinx individuals. Salas Pujols (2022) discusses counter spaces where conversations about Blackness were automatic and supportive. These spaces critiqued anti-Blackness and encouraged students to develop a critical consciousness. These spaces allowed Multiracial individuals to reflect on their racialized experiences and be more aware of their identity and positionality in the different dimensions of their lives. García-Louis and Cortes (2023) speak about how some students choose other aspects of their identity or “subculture,” such as centering other immigrant friends or those with similar interests and ideals. These affirming microsystems allowed students to affirm their ERI while offering positive experiences that countered the negative messages from family and school peers that rejected their Blackness.

### 4.2 Adult perspectives

The majority of the articles in this literature review (*n* = 13) come from the adult perspective, that is, Multiracial adults reflecting on their experiences growing up as a Multiracial child/adolescent. Three common themes emerged among these articles: (1) messages participants received, (2) how participants’ identity shifted across time and social contexts, and (3) how participants coped with identity-related challenges.

Messages participants reported receiving included both positive and negative messages from family, schools, and other communities they were a part of. Many common messages included family members making racialized comments regarding their body, such as their skin color or hair texture. Some family members offered ways to manage these features, such as telling them to stay out of the sun or to straighten their hair. In contrast, other family members encouraged Multiracial individuals to embrace their biracial ness and to be proud of their roots. For Multiracial adolescents who received conflicting messages from family members this created a feeling of cultural dissonance for them at the same time as they were in the process of learning to navigate the different aspects of their Multiracial identity.

One common occurrence for AfroLatinx individuals was the rejection of Blackness as part of Latinidad, meaning that Latinidad and Blackness were perceived as mutually exclusive, across their different communities. For Afrolatinx individuals, these messages influenced the way they viewed themselves and their belonging and membership to Blackness and Latinidad (Hordge-Freeman and Veras, 2020). On the other hand, some AfroLatinx individuals reported that they were told by grandparents to identify as Black, because African Americans have fought longer and harder and experienced less discrimination in the community (Romo, 2011).

A second theme that emerged from the research literature were the ways in which the identity shifted across time and contexts among Multiracial individuals. For example, Multiracial individuals developed a “AfroLatinidad” identity through exposure to their different communities (Hordge-Freeman and Veras, 2020). Furthermore, some Multiracial individuals shared how they negotiated the different aspects of their identity when their physical appearance contradicted the stereotypical representations of their ethnic-racial backgrounds (Romo, 2011). Multiracial individuals also recognized how they shifted their identity, if they believed they were perceived differently depending on where they live, and how their physical appearance might have changed over time, particularly if they became more racially ambiguous with age (Romo, 2011).

A different theme that arose related to identity was how Multiracial individuals with a white background identified. Across the articles, when a Multiracial individual had a White parent they were more likely to identify with their parent of color (i.e., Native, Asian) rather than with the white parent, except in cases where their phenotype was not aligned with the stereotypical image of a person of color (Khanna, 2004; Iwasaki and Byrd, 2010; Hordge-Freeman and Loblack, 2021; Strmic-Pawl et al., 2022).

Lastly, coping was a third theme that emerged from adult perspectives. Although Multiracial individuals reflected on the challenges they faced, they also recognized how they overcame and coped with these challenges. Iwasaki and Byrd (2010) shared how learning about heritage gave multiracial Native individuals a sense of peace and mental clarity, meaning they were in a state of peace both socially and personally. Having time to learn about the different aspects of their cultural heritage, as well as finding culturally affirming spaces (Jackson et al., 2013), including both in-person and online communities (Hordge-Freeman and Veras, 2020; McCoy Gay et al., 2022), enabled Multiracial individuals to make sense of their identity and place within society. This included learning about, accepting, and using new terms, such as “AfroLatinx” to describe themselves (Hordge-Freeman and Veras, 2020).

### 4.3 Parent perspectives

Lastly, three articles (*n* = 3) from the parent perspective were unique in that a parent of a Multiracial child(ren) shared how they viewed their child(ren)’s ERI. Kana’iaupuni and Liebler (2005) and Liebler (2010) looked at Census data to see how parents racially identified their child(ren). Kana’iaupuni and Liebler (2005) focused on the 1990 census and Multiracial Native Hawaiian classification to see how parents chose racial categories for their children when they were forced to choose. In a follow up study, Liebler (2010) focused on the 2000 census and Multiracial American Indian or Alaska Native in relation to homelands, since this was the first Census in which respondents could select more than one racial category. Kana’iaupuni and Liebler (2005) study suggests that physical and symbolic ties to Hawai’i are imperative to Hawaiian racial identification for multiracial children. Having ties to this cultural physical place (i.e., microsystem) increased Hawaiian racial identification. Hawaiian culture emphasizes physical and spiritual connection to land, the importance of genealogical family and ancestral ties, and the underpinning effects of colonization. Connection to these cultural notions played a significant role in racial identification for multiracial children. Multiracial children who had less cultural and physical connection to Hawai’i were found more often to assign the racial identity of their non-Hawaiian parents when required to report only one race.

Liebler (2010) found similar results to Kana’iaupuni and Liebler (2005), in which homelands play a significant role in single-race classification for Multiracial American Indians. In this article, homelands are considered a culturally meaningful physical space, typically the indigenous and ancestral homeland. Parents are more likely to report their child(ren) as being single-race American Indian if the American Indian parent reported a tribal affiliation and if an American Indian language is spoken at home. Parents may report their child(ren) as single-race non-American Indian or as multiracial if a non-English and non-American Indian language is reported at home and if they live in a racially segregated homeland. Liebler (2010) suggests that the biracial person may feel socially distant from families where everyone is a single-race American Indian, which may make them less likely to claim a single-race American Indian identity. These findings demonstrate the importance of physical space to the claimed ERI.

Kohli et al. (2020) utilized a qualitative approach to identify Asian Indian mothers who were raising Asian Indian and white Multiracial children to learn how these families adjusted their cultural values and practices. These mothers identified challenges their children faced such as navigating multiple cultures and racism. These challenges were unavoidable, but the mothers raised their children to be prepared for these types of challenges.

One unique finding from the Kohli et al. (2020) study was that these Asian Indian mothers wanted their children to explore and understand the different aspects of their identity and to identify with both cultures and racial groups. The mothers recognized the cultural dissonance of navigating these different cultures, especially when in majority-white spaces. Having a non-Asian Indian father was a way for children to learn how to navigate these spaces making it easier to understand Eurocentric social norms and expectations. The mothers also acknowledged difficulties Multiracial children have in developing their identity. For example, some children shifted from an Asian-Indian identity to a white identity to fit in at their majority-white high school. Along with struggling with their sense of belonging in school, children experienced stereotypes, such as being good at science and math. Although these stereotypes appear positive, such stereotypes arouse mixed feelings and produce negative consequences since this broad abstraction may not apply to all individuals in this ethnic-racial group (Ramasubramanian et al., 2020). Furthermore, other scholars have suggested that positive stereotypes come from Eurocentric ideals, thus raising internal identity conflicts (Garay and Remedios, 2021).

Kohli et al. (2020) emphasized that the mothers recognized that their Multiracial children possessed unique strengths and assets, and were more self-reflective, resilient, adaptive, and willing to stand up for themselves. Beyond just bringing diversity to the spaces they occupy, parents felt that their children had a sense of pride and responsibility for teaching others about their racial and cultural backgrounds. The finding suggests that even though Multiracial children face unique challenges and barriers, they can overcome such challenges and transform the spaces they inhabit to positive spaces.

## 5 Discussion

This systematic literature review considered how Multiracial youth develop and reconcile their identity, while considering how messages from family members and peers influence their identity development. Research with adolescents emphasized the importance of different spaces (i.e., home, school, community) that influenced their racial identity development while studies with Multiracial adults revealed how messages, verbal and/or nonverbal, received from different sources, influenced them to tryout and/or shift racial identity over time as they matured and expanded their repertoire of situational contexts. Finally, older respondents described how they coped with unique stressors tied to their racial identity and whether the messages were positive or negative. Lastly, the parent perspective offered a unique understanding of how parents recognized the challenges their children face and how parents sought to enable their Multiracial children to overcome challenges while creating a more diverse and inclusive world. Particularly problematic was the situation where mono-racial parent(s) tried to socialize their multiracial children into a world that they themselves had not had to experience.

Microsystems, including family systems, schools, affinity groups, and homelands, all seem to be important socializers and influencers to claiming or rejecting a Multiracial identity. Oftentimes, these microsystems provided mixed and conflicting messages about belonging to different ethnic-racial groups. For example, for some Latinx individuals, family members spoke disparagingly of the youth’s Black identity and experience, while at the same time Latinx peers invalidated their Latinidad as insufficient. Thus, biracial individuals face the inevitable reality that often their two different microsystems clash in their acceptance or rejection of their different identities. However, some individuals reported finding other supportive microsystems that affirmed their dual identities and strengthened any feeling of belonging to two racial groups (Salas Pujols, 2022; García-Louis and Cortes, 2023). Microsystems outside of the sphere of the family played an invaluable role in helping individuals with their biracial identity development while at the same time being influential in how parents identify their children. Importantly, having a physical, socio-cultural, or spiritual connection to a microsystem influenced the way an individual identified racially, and how others identified them.

Furthermore, the studies included in this review highlight the unique internal assets and external resources that Multiracial individuals utilize (Benson, 2007; Search Institute, 2011). Consistent internal assets include learning and being reflective about the different aspects of identity and learning positive ways for coping with microaggressions. Consistent external resources included support from family, peers, and community related programs and spaces that reinforced Multiracial youth’s quest for inclusion of the different aspects of their biracial identity.

One unique aspect from this literature review is that research highlights the fact that ethnic-racial identity is a continuous lifelong process and does not share the stability that monoracial individuals enjoy with their racial identity. One highlight from the literature is how some Multiracial individuals shifted identity, based on changes in their physical appearance (Khanna, 2004) or and the degree to which they tried to fit in with their peers (Kohli et al., 2020). Furthermore, racial identity may change once the individual has the opportunity to explore different aspects of their identity within their different communities (Salas Pujols, 2022). This would be akin to developing a sense of cultural and psychological belonging to both ethnic-racial orientations; or what is often called biculturalism (LaFromboise et al., 1993) in the literature.

## 6 Directions for future research

The studies that met the criteria for inclusion in this review of literature hint at the field’s limitations beginning with the scarcity of research on Multiracial children, especially among groups other than Black/White mixed race children. Beyond this is the scarcity of research that examines how Multiracial adolescents frame their identity depending on the gender of the parent of color, the racial/ethnic density of the community and schools they attend, their friendship networks and whether these include other Multiracial children/adolescents, supportive relationships with non-parental family members and the efforts taken by both parents to socialize children into the racial realities of the community and society at large. Future research should focus on centering youth voices, specifically non-Black/white biracial youth, especially when examining developmental influences from different ecological levels. Research should also specify the Multiracial backgrounds of the participants. Even though Multiracial individuals share similar experiences, their unique racial, heritage and cultural backgrounds offer unique insights into how Multiracial individuals are accepted or distanced depending on such considerations as phenotype, parents’ social class, and a community’s social and political tolerance for Multiracial diversity that goes beyond the verbal, but which can be witnessed by an examination of residential housing patterns that may suggest some segregation that determines public school enrollments and achievement outcomes. Also important is the social messaging in a community (and school) that is affirming of diversity and where Multiracial children and youth feel safe and experience positive feelings of belonging.

## Data availability statement

The original contributions presented in the study are included in the article/supplementary material, further inquiries can be directed to the corresponding author.

## Author contributions

TZ: Writing – review & editing, Writing – original draft. AP: Writing – review & editing.
